# Altered Kv2.1 functioning promotes increased excitability in hippocampal neurons of an Alzheimer's disease mouse model

**DOI:** 10.1038/cddis.2016.18

**Published:** 2016-02-18

**Authors:** V Frazzini, S Guarnieri, M Bomba, R Navarra, C Morabito, M A Mariggiò, S L Sensi

**Affiliations:** 1 Molecular Neurology Unit, Center of Excellence on Aging and Translational Medicine (CeSI-MeT), University “G. d'Annunzio”, Chieti-Pescara, Chieti, Italy; 2 Department of Neuroscience and Clinical Sciences, University “G. d'Annunzio”, Chieti, Italy; 3 Cellular Physiology Unit, CeSI-MeT, University “G. d'Annunzio”, Chieti-Pescara, Chieti, Italy; 4 Departments of Neurology and Pharmacology, Institute for Memory Impairments and Neurological Disorders, University of California-Irvine, Irvine, CA, USA

## Abstract

Altered neuronal excitability is emerging as an important feature in Alzheimer's disease (AD). Kv2.1 potassium channels are important modulators of neuronal excitability and synaptic activity. We investigated Kv2.1 currents and its relation to the intrinsic synaptic activity of hippocampal neurons from 3xTg-AD (triple transgenic mouse model of Alzheimer's disease) mice, a widely employed preclinical AD model. Synaptic activity was also investigated by analyzing spontaneous [Ca^2+^]_i_ spikes. Compared with wild-type (Non-Tg (non-transgenic mouse model)) cultures, 3xTg-AD neurons showed enhanced spike frequency and decreased intensity. Compared with Non-Tg cultures, 3xTg-AD hippocampal neurons revealed reduced Kv2.1-dependent *I*_k_ current densities as well as normalized conductances. 3xTg-AD cultures also exhibited an overall decrease in the number of functional Kv2.1 channels. Immunofluorescence assay revealed an increase in Kv2.1 channel oligomerization, a condition associated with blockade of channel function. In Non-Tg neurons, pharmacological blockade of Kv2.1 channels reproduced the altered pattern found in the 3xTg-AD cultures. Moreover, compared with untreated sister cultures, pharmacological inhibition of Kv2.1 in 3xTg-AD neurons did not produce any significant modification in *I*_k_ current densities. Reactive oxygen species (ROS) promote Kv2.1 oligomerization, thereby acting as negative modulator of the channel activity. Glutamate receptor activation produced higher ROS levels in hippocampal 3xTg-AD cultures compared with Non-Tg neurons. Antioxidant treatment with *N*-Acetyl-Cysteine was found to rescue Kv2.1-dependent currents and decreased spontaneous hyperexcitability in 3xTg-AD neurons. Analogous results regarding spontaneous synaptic activity were observed in neuronal cultures treated with the antioxidant 6-hydroxy-2,5,7,8-tetramethylchroman-2-carboxylic acid (Trolox). Our study indicates that AD-related mutations may promote enhanced ROS generation, oxidative-dependent oligomerization, and loss of function of Kv2.1 channels. These processes can be part on the increased neuronal excitability of these neurons. These steps may set a deleterious vicious circle that eventually helps to promote excitotoxic damage found in the AD brain.

Alzheimer's disease (AD) is the most common cause of dementia in the elderly. Among other mechanisms, dementia of AD type can result from progressive functional and structural derangement of synapses, dendrites, brain circuits, nodes and networks.^1, 2^

According to recent findings, the AD-related deregulation of brain activity encompasses the simultaneous presence of hyperactive and hypoactive neurons that reorganize their activity in functional clusters.^3^ This reorganization ultimately contributes to the production of network hyperexcitability and instability, hypersynchrony, epileptiform spiking as well as the activation of compensatory circuit remodeling.^4, 5^

At the neuronal level, the homeostatic regulation of excitability and plasticity relies on the coordinated functioning of a wide array of ionic channels.^6^ In that context, delayed rectifier K^+^ currents (*I*_K_) have a major role in modulating cellular excitability. Kv2.1 channels are one of the most widely expressed Kv channels in the brain and considered the main determinants of *I*_K_ currents in hippocampal neurons.^7, 8^ Kv2.1 channels control excitability but also have an important role in neuronal apoptosis.^9, 10^ Kv2.1 channels show very slow activation kinetics and deactivation dynamics, preventing complete channel deactivation upon sustained neuronal activity.

Oxidative stress has been shown to act as negative modulator of Kv2.1-dependent K^+^ currents,^11, 12^ an intriguing finding considering the crucial role had by oxidative stress in the early stages of AD.^13, 14^

Considering the AD-related hyperexcitability and oxidative stress, it is possible that the Kv2.1 channel oxidation that may occur early on in the disease can promote unbalanced neuronal excitability and activity-dependent regulation as well as network instability and dysfunction. With this conceptual framework as reference, we studied the potential relationship occurring between changes in the levels of oxidative stress, alterations of Kv2.1-dependent K^+^ currents, and variations in neuronal intrinsic activity in hippocampal neurons obtained from 3xTg-AD (triple transgenic mouse model of Alzheimer's disease) mice. The 3xTg-AD mouse, a widely investigated preclinical model of AD, has the benefit of exhibiting an age-related development of *β*-Amyloid (A*β*)- and tau-dependent pathology as well as AD-related synaptic dysfunction and cognitive impairment.^15^

## Results

### 3xTg-AD hippocampal neurons show increased [Ca^2+^]_i_ spike frequency

Spontaneous activity is an important determinant of information processing in neuronal circuits and in the brain.^5, 16, 17, 18^ Neurons develop spontaneous rhythmic and bursting activity at the end of the first week in culture,^19, 20^ a property that has been exploited to investigate basic aspects of network functioning *in vitro*.

**Figure 1:**
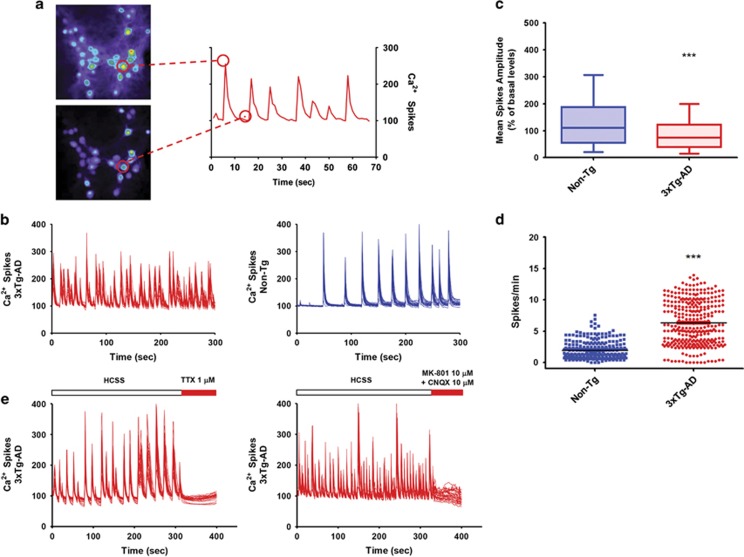
3xTg-AD hippocampal neurons show increased [Ca^2+^]_i_ spike frequency compared with Non-Tg cultures. (**a**) Representative spontaneous Ca^2+^ spike activity occurring in 3xTg-AD cultured hippocampal neurons. Left panel shows pseudocolor images of Fluo-4-loaded cultures. Right panel shows time courses of Ca^2+^ transients occurring in neurons identified by the circle on the left panel. (**b**) Representative time course traces of spontaneous spike activity occurring in 3xTg-AD and Non-Tg cultures. (**c**) Plot graph depicts averaged Ca^2+^ spike amplitudes in 3xTg-AD (average values of 321 neurons from 14 independent experiments) and Non-Tg cultures (average values of 321 neurons from 15 independent experiments; *P*<0.001; unpaired *t*-test). (**d**) Dot plot shows averaged spike frequencies in 3xTg-AD (322 neurons from 14 independent experiments) and Non-Tg cultures (321 neurons from 15 independent experiments). Note the reduced frequency in the 3xTg-AD population (*P*<0.001; unpaired *t*-test). (**e**) Representative time course of spike activity occurring in 3xTg-AD cultures treated with TTX (1 *μ*M) or glutamate receptor antagonists (MK-801 (10 *μ*M) and CNQX (10 *μ*M)). Note that both treatments produced strong inhibition of spontaneous synaptic activity

[Ca^2+^]_i_ transients are considered an indirect index of the action potential firing status.^21, 22^ With real-time microfluorimetry, we evaluated spontaneous [Ca^2+^]_i_ transients occurring in soma of 3xTg-AD or Non-Tg (non-transgenic mouse model) hippocampal neurons. Hippocampal neurons were loaded with the high affinity Ca^2+^ sensitive probe Fluo-4 AM and transients assessed in terms of number of spikes for minute (Figure 1a and b). In Non-Tg neurons, the pattern of spontaneous activity was found to be remarkably homogenous among different clusters (average spike frequency values were 1.958±0.073 (S.E.M.) in 321 neurons from 15 dishes; Figures 1b and d). Compared with Non-Tg cells, 3xTg-AD neurons showed higher spike frequency (average spike frequency values were 6.312±0.1965 (S.E.M.) in 322 neurons from 14 cultures; *P*<0.001; Figure 1b and d). In 3xTg-AD neurons, increased Ca^2+^ spike frequency was associated with lower average spike intensity (average fluorescence values were 85.71±3.165 (S.E.M.) 321 neurons from 14 cultures; Figure 1c) compared with Non-Tg (mean fluorescence values: 131.4±4.986 (S.E.M.) from 321 neurons from 15 cultures; *P*<0.001; Figure 1c). Treatment with the sodium channel blocker, tetrodotoxin (TTX), completely blocked [Ca^2+^]_i_ transients, thereby indicating that the observed oscillations were dependent on action potential firing. Moreover, transients were also completely abolished by the application of the ionotropic glutamate receptor blockers CNQX (6-cyano-7-nitroquinoxaline-2,3-dione; 10 *μ*M) and MK-801 (10 *μ*M), thereby demonstrating that increased [Ca^2+^]_i_ spike frequencies depend on enhanced spontaneous synaptic activity mediated by glutamate release and receptor activation (Figure 1e).

### 3xTg-AD mice show decreased delayed rectifier current density

In hippocampal neurons, Kv2.1 channels are the main driver for delayed rectifier K^+^ currents (*I*_k_).^7, 23^ The channels negatively modulate neuronal activity during repetitive spiking and promote neuronal integration by suppressing hyperexcitability when repetitive signals approach the soma. Kv2.1 channels undergo an oxidative-dependent loss of function, a phenomenon that may promote the increased spontaneous activity that we have observed in 3xTg-AD neurons. Thus, employing patch-clamp whole-cell recordings, we investigated Kv2.1-dependent currents in our 3xTg-AD and Non-Tg hippocampal neurons.

At first, we assessed if this baseline shift of membrane potential was present in our Tg-AD cultures and found no statistical significant differences in resting membrane potentials values between 3xTg-AD and Non-Tg neurons (Supplementary Figure 1A).

**Figure 2:**
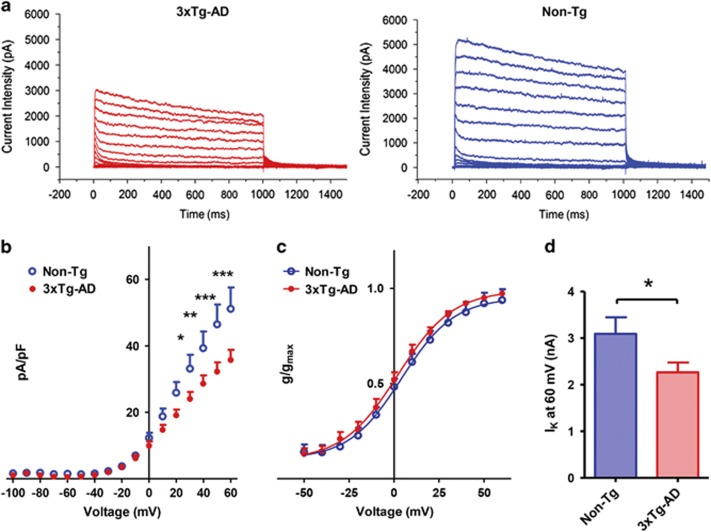
3xTg-AD mice show decreased delayed rectifier current density. (**a**) Representative *I*_k_ currents recorded from 3xTg-AD (left traces) and Non-Tg neurons (right traces). (**b**) *I*/*V* plot of *I*_k_ currents in 3xTg-AD and Non-Tg cultures. Graph shows normalized current intensities from 3xTg-AD (*n*=17) and Non-Tg neurons (*n*=10). Note that, compared with Non-Tg, 3xTg-AD neurons show decreased Kv2.1-dependent current densities (*P<0.05; **P<0.01; ***P<0.001; two-way ANOVA). (**c**) Plot shows activation curves from 3xTg-AD and Non-Tg neurons. Note that the residual channel population is not significantly different in the two strains. (**d**) Bar graph depicts averaged peak current intensity values of 17 3xTg-AD and 10 Non-Tg neurons. Note that, compared with Non-Tg, 3xTg-AD cells displayed lower peak current intensities at +60 mV (*P*<0.05; unpaired *t*-test)

Kv2.1-dependent current density values were instead found to be significantly lower in 3xTg-AD neurons (average current density values (pA/pF) recorded from 17 neurons were 24.066±2.061 (S.E.M.) at +30 mV, 28.632±2.48 (S.E.M.) at +40 mV, 32.258±2.774 at +50 mV and 35.770±3.064 (S.E.M.) +60 mV; Figure 2a and b) compared with Non-Tg cells (average pA/pF values in 10 neurons were 33.147±4.171 (S.E.M.) at +30 mV (*P*<0.05), 39.331±5.008 (S.E.M.) at +40 mV (*P*<0.01), 46.503±5.961 at +50 mV (*P*<0.001) and 51.151±6.448 at +60 mV (*P*<0.001); Figure 2a and b). Moreover, 3xTg-AD neurons displayed lower peaks of current intensities recorded at +60 mV (average current intensity (nA) values: 2.266±0.2114 (S.E.M.) from 17 neurons; Figure 2d) compared with Non-Tg (average nA values: 3.090±0.358 (S.E.M.) from 10 neurons (*P*<0.05); Figure 2d). Analysis of current activation kinetics did not show differences between the two strains (Figure 2c).

Taken together, these findings indicate that 3xTg-AD neurons express a lower number of fully functional Kv2.1 channels on their plasma membranes. No biophysical differences were found in the residual channel population between Non-Tg and 3xTg-AD neurons.

### 3xTg-AD hippocampal neurons show increased ROS production triggered by NMDA receptor activation

**Figure 3:**
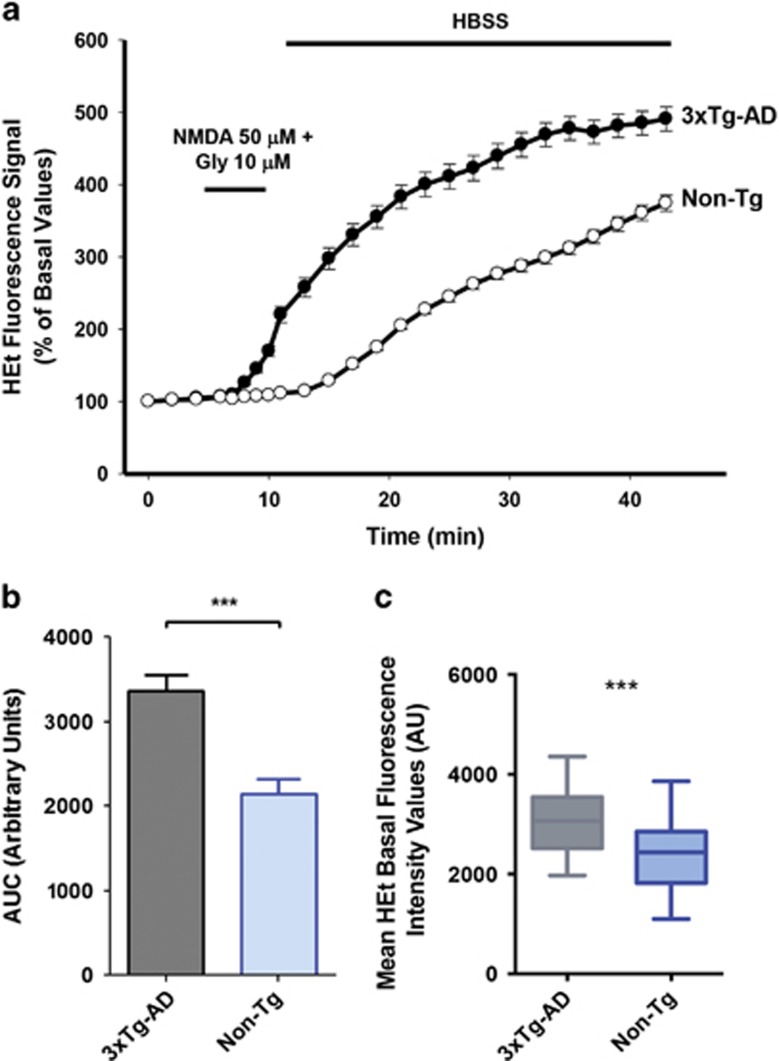
3xTg-AD hippocampal neurons show increased ROS production upon NMDA exposure. (**a**) Traces show representative HEt fluorescence changes over time in neurons from 3xTg-AD (black circles) or Non-Tg (white circles) neurons exposed for 5 min to NMDA (50 *μ*M)+glycine (10 *μ*M). (**b**) Bar graph shows average cumulative changes in HEt fluorescence expressed as area under the curve (AUC) in Non-Tg (108 neurons from five independent experiments) and 3xTg-AD cultures (145 neurons from five independent experiments). Note that, compared with Non-Tg cultures, 3xTg-AD neurons show higher ROS production upon NMDA exposure (*P*<0.001; unpaired *t*-test). (**c**) Plot graph depicts average basal fluorescence values detected in 3xTg-AD neurons (291 cells from five experiments) and Non-Tg neurons (295 neurons from five experiments). Compared with Non-Tg, 3xTg-AD neurons show higher baseline Het fluorescence values (***P<0.001, unpaired *t*-test)

Moderate levels of ROS production have been shown to be important modulators of neuronal signaling.^24, 25^ ROS overproduction promotes damage by acting on multiple intracellular targets. To determine whether the reduction in macroscopic currents that we have observed in 3xTg-AD cultures is linked to increased ROS production and subsequent ROS-dependent loss of function of Kv2.1 channels, we examined cytosolic ROS levels evoked by a mild excitotoxic challenge obtained with exposure to *N*-methyl-d-aspartate (NMDA). To that aim, hydroethidine (HEt)-loaded neurons were challenged for 5 min with NMDA (50 *μ*M, in presence of 10 *μ*M of the receptor co-activator glycine; Figure 3a). Compared twith Non-Tg cultures (where average fluorescence values were 2146±173.6 (S.E.M.) in 108 neurons from 5 cultures; Figure 3b), 3xTg-AD neurons showed higher ROS production (average fluorescence values: 3366±188.4 (S.E.M.) in 145 neurons from 5 cultures; *P*<0.001; Figure 3b). These data indicate that, upon mild excitotoxic conditions, 3xTg-AD neuronal cultures may be more prone to ROS production. To evaluate baseline levels of oxidative stress occurring in 3xTg-AD and Non-Tg neurons, we then analyzed the culture basal ROS levels by assessing differences in HEt fluorescence values. With this approach, changes in baseline fluorescence values are representative of the undergoing cumulative variations in ROS production. Results of this set of experiments indicate that 3xTg-AD neurons show statistically significant (*P*<0.001, Figure 3c) higher ROS levels at baseline (average fluorescence values: 3080±42,28, (S.E.M.) in 291 neurons from 5 cultures; Figure 3c) compared with Non-Tg neurons (average fluorescence values: 2389±48,17 (S.E.M.) in 295 neurons from 5 cultures).

### 3xTg-AD hippocampal neurons display increased expression and clusterization of Kv2.1 channels on somato-dendritic membranes

**Figure 4:**
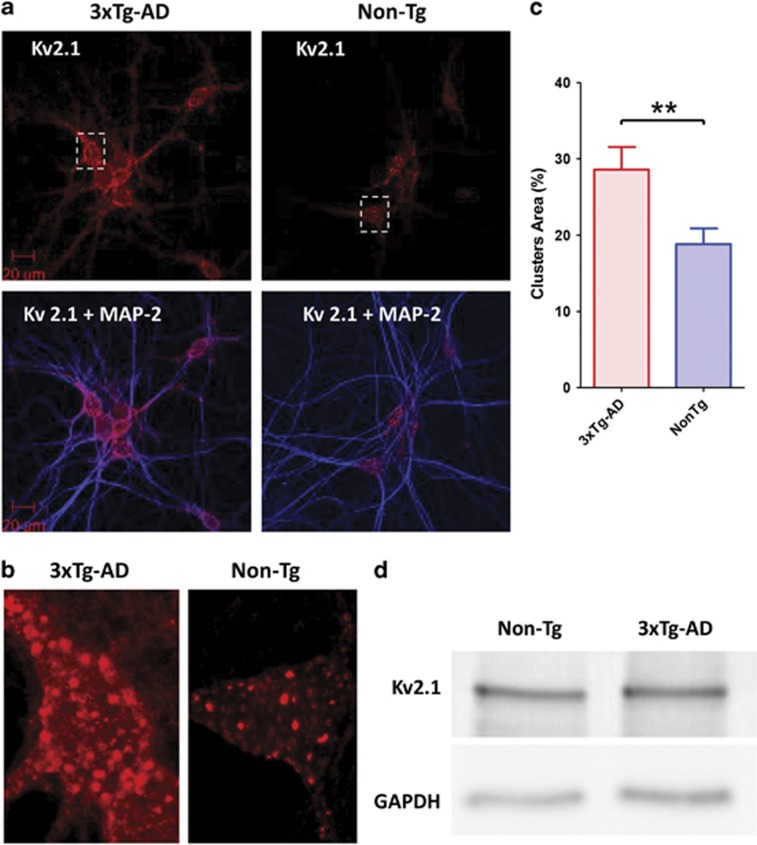
3xTg-AD hippocampal neurons display increased expression of Kv2.1 channels. (**a**) Representative confocal images of hippocampal neurons from 3xTg-AD (left panels) and Non-Tg cultures (right panels) stained with anti-Kv2.1 (red, top panels) or anti-Kv2.1 and anti-MAP-2 (red and blue; lower panels) antibodies. (**b**) Magnification of neuronal somata from the confocal images shown in the upper panels of **a**. (**c**) Bar graph depicts quantification of the cluster area (expressed as % of somatic areas occupied by the Kv2.1 clusters) in 3xTg-AD (*n*=26) and Non-Tg (*n*=36) neurons. Note the statistically significant higher clusterization of 3xTg-AD cultures (*P*<0.01; unpaired *t*-test). (**d**) Kv2.1 immunoblotting indicates no differences in channel expression in the two strains

Oxidized Kv2.1 channels assemble in oligomers that are held together by disulfide bridges involving Cys-73.^12^ The oligomerization process has been shown to lead to progressive accumulation of dysfunctional aggregates on neuronal plasma membranes.^11, 12^ To evaluate whether chronic oxidative stress induces Kv2.1 channel oligomerization in our AD model, hippocampal neurons were fixed and stained with a Kv2.1 antibody and clustering analysis performed. As expected, Kv2.1 channel expression was found to be strongly restricted to neuronal soma and proximal dendrites. When compared with Non-Tg cultures, 3xTg-AD neurons showed an overall increase in channel localization on neuronal membranes (Figure 4a and b). For each neuron, we measured the surface area occupied by Kv2.1 clusters within a defined somatic region, thereby obtaining a parametric ‘clusterization' index. Thus, the index represents the fraction of the total plasma membrane area that is occupied by, immunohistochemically identified, Kv2.1 channels. 3xTg-AD neurons showed higher clusterization (average clusterization area in % was 28.58±2.963 (S.E.M.) in 26 neurons) compared with Non-Tg neurons (average clusterization area in % was 18.82±2.061 (S.E.M.) in 36 neurons; *P*<0.01). A previous *in vivo* study has shown that A*β* oligomers can modify Kv2.1 expression and promote increased channel synthesis.^26^ Moreover, increased expression has also been shown to enhance channel clusterization.^27^ To assess whether the increased Kv2.1 clusterization that we found in our AD model is dependent on higher levels of channel conglomeration but not synthesis, we performed a Kv2.1 western blot analysis in the two strains. WB results indicated overlapping amounts of Kv2.1 channels in the two cultures (Figure 4c).

Interestingly, 3xTg-AD hippocampal neurons exposed to an excitotoxic-like condition showed Kv.2.1 de-clustering. This finding suggests that, upon prolonged glutamatergic stimulation, 3xTg-AD neurons are, most likely, employing homeostatic mechanisms to regain basal levels of Kv2.1-mediated excitability (Supplementary Figure 1B and C).

In summary, our Ca^2+^ imaging and electrophysiological experiments support the idea that 3xTg-AD neurons may develop a progressive accumulation of conglomerates of dysfunctional channels on their neuronal surfaces.

### Pharmacological blockade of Kv2.1 channels promotes I_k_ decrease and [Ca^2+^]_i_ spike frequency enhancement in Non-Tg neurons and has no effect in 3xTg-AD neurons

**Figure 5:**
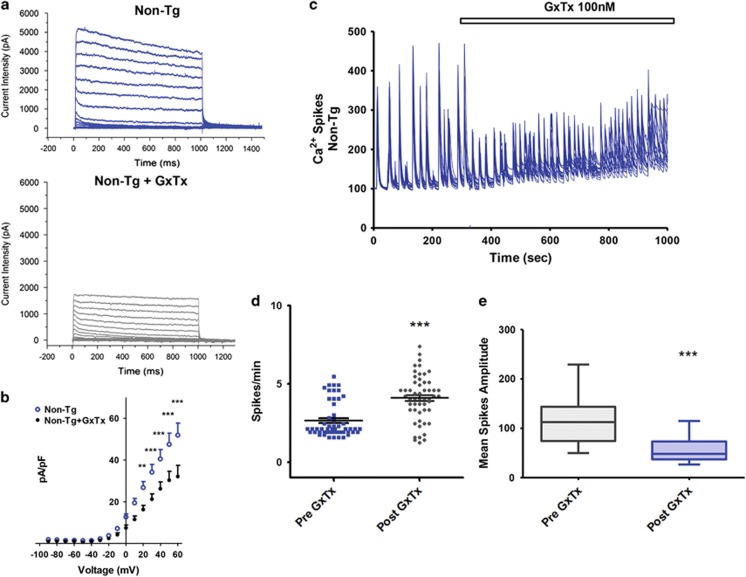
Pharmacological blockade of Kv2.1 decreases *I*_K_ and enhances [Ca^2+^]_i_ spike frequency in Non-Tg neurons. (**a**) Representative traces of *I*_k_ current density in untreated (upper trace) and GxTx-treated (100 nM) Non-Tg neurons (lower trace). (**b**) *I*/*V* plot of *I*_k_ currents in treated and untreated Non-Tg cultures. Graph shows normalized current intensities from 10 untreated and 6 treated Non-Tg neurons. Note that GxTx-treated neurons show a decrement in *I*_k_ currents when compared with untreated sister cultures (***P<0.001; **P<0.01; *P<0.05; two-way ANOVA). (**c**) Representative traces of spontaneous [Ca^2+^]_i_ spike activity in Non-Tg neurons before and after application of (100 nM) GxTx. (**d**) Dot plot graph depicts quantification of spike frequencies found in Non-Tg cultures before and after GxTx application (59 neurons from three dishes). Note the increased spike frequency occurring after GxTx exposure (*P*<0.001; unpaired *t*-test). (**e**) Plot graph depicts averaged spike amplitudes in Non-Tg neurons before (mean fluorescence values: 118.6±7.104 (S.E.M.) from 59 neurons of three cultures) and after GxTx application (average fluorescence values: 58.03±3.771 (S.E.M.) from the same 59 neurons; *P*<0.001; unpaired *t*-test)

**Figure 6:**
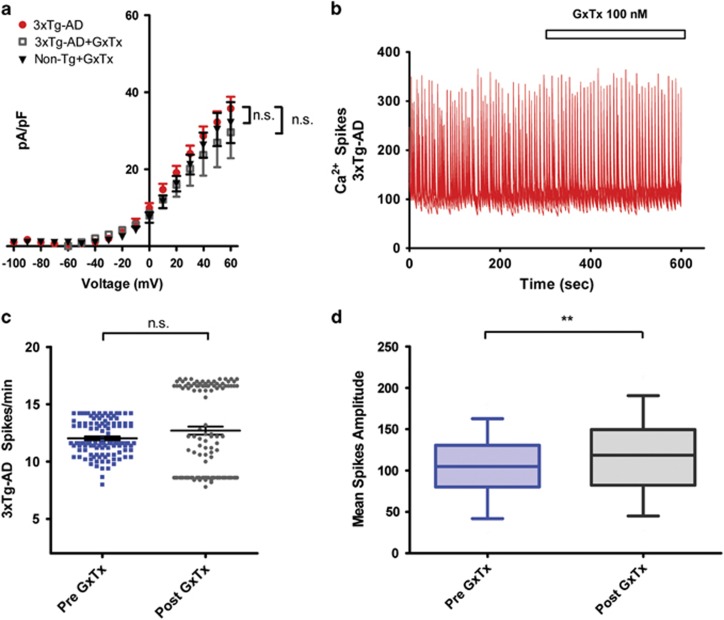
Pharmacological blockade of Kv2.1 does not affect *I*_k_ currents and [Ca^2+^]_i_ spike frequency in 3xTg-AD neurons. (**a**) *I*/*V* plot of *I*_k_ currents in untreated and GxTx-treated (100 nM) 3xTg-AD neurons and from untreated Non-Tg cultures. Graph shows normalized current intensities from 17 untreated 3xTg-AD neurons, 5 GxTx-treated 3xTg-AD neurons and 10 untreated Non-Tg neurons. Note that GxTx-treated neurons did not show any *I*_k_ current modifications when compared with untreated sister cultures (*P*>0.05; two-way ANOVA). (**b**) Representative traces of spontaneous [Ca^2+^]_i_ spike activity in 3xTg-AD neurons before and after application of (100 nM) GxTx. (**c**) Dot plot graph depicts quantification of spike frequencies found in 3xTg-AD cultures before and after GxTx application (113 neurons from three dishes). Note the absence of spike frequency modification that occurs after GxTx exposure (*P*>0.05; unpaired *t*-test). (**d**) Plot graph depicts average spike amplitudes in Non-Tg neurons before (average fluorescence values: 105.0±3.380 (S.E.M.) from 113 neurons from 3 cultures) and after GxTx application (average fluorescence values: 119.5±4.207 (S.E.M.) from the same 113 neurons; *P*<0.01; unpaired *t*-test)

To evaluate whether the loss of function of Kv2.1 channels that we have found in the 3xTg-AD cultures is functionally related to the observed increased excitability, we performed complementary pharmacological manipulations on Non-Tg neurons in order to induce and mimic the behavior showed by 3xTg-AD neurons. To that aim, Non-Tg cultures were treated with the Kv2.1 channel blocker Guangxitoxin (GxTx) and whole-cell *I*_k_ currents recorded. GxTx is a gating modifier of Kv2.1 channels (with a IC_50_ of 1 nM for Kv2.1, 3 nM for Kv2.2 and 50 nM for Kv4.3 and no significant effects on other voltage gated ion channels). Non-Tg neurons were treated with GxTx (100 nM) throughout the recordings. GxTx-treated neurons showed *I*_k_ currents that were decreased (average pA/pF values recorded from 6 neurons were 21.216±2.569 (S.E.M.) at +30 mV; 26.256±3.241 (S.E.M.) at +40 mV; 30.315±4.330 (S.E.M.) at +50 mV and 32.117±5.314 (S.E.M.) at +60 mV; Figure 5a and b) compared with untreated sister cultures (average pA/pF values recorded from 10 neurons were 36.227±3.403 (S.E.M.) at +30 mV, (*P*<0.05); 42.990±4.200 (S.E.M.) at +40 mV, (*P*<0.001); 50.489±4.936 (S.E.M.) at +50 mV, (*P*<0.001) and 55.194±5.296 (S.E.M.) at +60 mV, (*P*<0.001); Figure 5a and b). GxTx effects were also evaluated on spontaneous [Ca^2+^]_i_ spikes of Non-Tg neurons. GxTx, applied throughout the whole imaging experiment, induced a significant increase in spike frequency (average spike frequency values: 4.361±0.1653 (S.E.M.) in 56 neurons from 3 dishes; *P*<0.001; Figure 5c and d) when compared with pre-drug basal values (average spike frequency values: 2.506±0.1400 (S.E.M.) in 59 neurons from three dishes; Figure 5c and d). Moreover, GxTx application also produced decreases in [Ca^2+^]_i_ amplitudes that resembled those observed in 3xTg-AD neurons (average fluorescence values in Non-Tg before GxTx challenge were 118.6±7.104 (S.E.M.) in 59 neurons from three cultures; average fluorescence values in Non-Tg after the GxTx challenge were 58.03±3.771 (S.E.M.) in 59 neurons from three cultures; *P*<0.001; Figure 5e). We also replicated Figure 5 experiments on 3xTg-AD neurons. GxTx-treated 3xTg-AD neurons (five neurons from three different cultures) did not show any *I*_k_ currents modifications when compared with untreated 3xTg-AD cells (17 neurons from three different cultures; *P*>0.05; Figure 6a). Moreover, no differences were found when GxTx-treated 3xTg-AD neurons were compared with GxTx-treated Non-Tg neurons (six neurons from three different cultures; *P*>0.05 for all voltage membrane tests; Figure 6a). Spontaneous [Ca^2+^]_i_ spike frequency in 3xTg-AD neurons was also similar before (average spike frequency value: 12.03±0.1424 (S.E.M.) in 113 neurons from three cultures) and after application of GxTx (100 nM) (spike frequency value: 12.70±0.3494 (S.E.M.) in 113 neurons from the same three cultures; *P*>0,05; Figure 6b and c). The finding that GxTx application does not modify what is naturally occurring in 3xTg-AD neurons corroborates the hypothesis that most of the neuron hyperexcitability is the results of dysfunctional Kv2.1 activity. However, analysis of amplitudes of spontaneous [Ca^2+^]_i_ spikes in GxTx-treated 3xTg-AD neurons revealed small but statistically significant increased values (average amplitude value: 119.5±4.207 (S.E.M.) in 113 neurons from three cultures) compared with those obtained in the same neurons before treatment (average fluorescence value was 105.0±3.380 (S.E.M.) from the same 113 neurons; *P*<0,01; Figure 6d).

### Antioxidant treatment decreases spontaneous synaptic activity in 3xTg-AD mice

**Figure 7:**
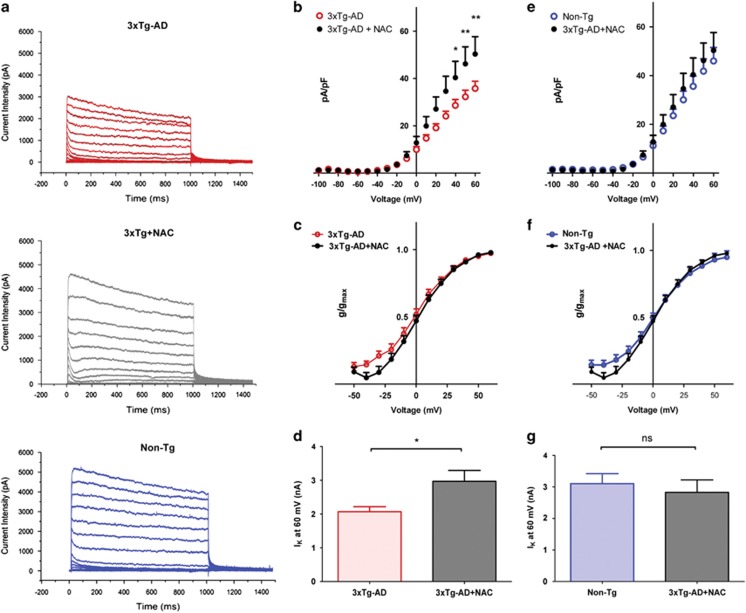
NAC treatment increases Kv2.1-dependent current densities in 3xTg-AD neurons. (**a**) Representative traces of *I*_k_ current density recorded from 3xTg-AD cultures (upper traces), 3xTg-AD cultures treated with NAC (1 mM; middle traces), and Non-Tg cultures (lower traces). (**b**) *I*/*V* plot of *I*_k_ currents. Graph depicts normalized current intensities of 17 untreated or 13 NAC-treated 3xTg-AD neurons (**P<0.01, *P<0.05; two-way ANOVA). (**c**) Plot shows activation curves from the same set of neurons shown in **b**. (**d**) Bar graph shows averaged peak currents values from the experiments shown in **b**. (**e**) *I*/*V* plot of *I*_k_ currents of 10 Non-Tg and 13 3xTg-AD+NAC neurons. Note that the trace shows non-statistically significant differences. (**f**) Plot shows activation curves from the same set of neurons shown in **e**. (**g**) Bar graph shows averaged peak current values from the experiments shown in **e**

**Figure 8:**
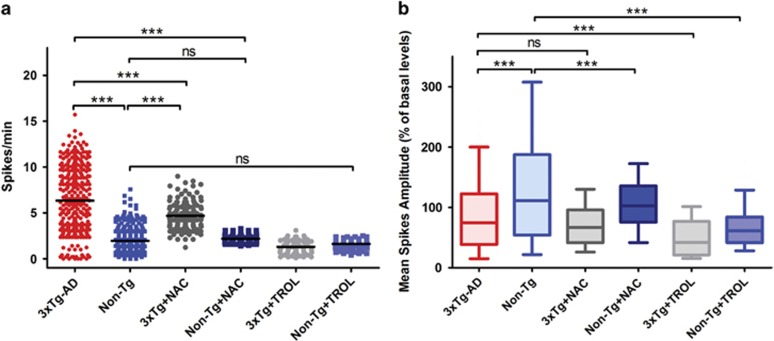
NAC and Trolox treatment decreases spontaneous synaptic activity in 3xTg-AD mice. (**a**) Dot plot graph depicts quantification of spike frequencies in untreated 3xTg-AD and NAC- or Trolox-treated 3xTg-AD neurons (321 untreated 3xTg-AD cells from 14 cultures; 143 NAC-treated cells from 7 cultures; 74 Trolox-treated cells from 3 cultures) along with untreated and NAC- or Trolox-treated Non-Tg neurons (231 untreated Non-Tg cells from 15 cultures; 231 NAC-treated cells from 7 cultures; 127 Trolox-treated cells from 4 cultures). Note that NAC treatment induced significant changes (***P<0.001; ANOVA and *post hoc* Bonferroni) only in 3xTg-AD cells whereas no effects were seen in Non-Tg cultures, thereby suggesting a selective role of oxidative stress in the modulation of 3xTg-AD neuronal activity. Similar effects are observed in Trolox-treated cultures. Trolox induced significant changes (***P<0.001; ANOVA and *post hoc* Bonferroni) in 3xTg-AD spiking activity while no effects are seen in Non-Tg cells. (**b**) Plot graph depicts averaged Ca^2+^ spike amplitudes relative to the same six experimental conditions shown in **a**. Note that NAC or Trolox does not induce significant changes in 3xTg-AD cells whereas the two molecules have significant effects in Non-Tg neurons (***P<0.001; ANOVA and *post hoc* Bonferroni)

In order to dissect the role of ROS in the modulation of Kv2.1-dependent currents in 3xTg-AD, we preincubated for 48 h 3xTg-AD neurons with the antioxidant compound, *N*-acetyl-cysteine (NAC), and assessed *I*_k_ currents. Compared with untreated cultures [average pA/pF values recorded from 17 neurons: 28.632±2.486 (S.E.M.) at +40 mV; 32.258±2.774 (S.E.M.) at +50 mV; 35.770±3.064 (S.E.M.) at +60 mV Figure 7a and b), NAC-treated cells displayed a significant rescue of Kv2.1-dependent current densities (average pA/pF values recorded from 12 neurons were 40.355±6.866 (S.E.M.) at +40 mV, *P*<0.05; at +50 mV: 46.195±7.192 (S.E.M.), *P*<0.01; at +60 mV: 50.334±7.354 (S.E.M.), *P*<0.01; Figure 7a and b) as well as of peak current values (mean nA values at +60 mV: 2.071±0.1457 (S.E.M.) from 15 3xTg-AD neurons; 2.970±0.3230 (S.E.M.) from 12 NAC-treated 3xTg-AD neurons; *P*< 0.05; Figure 7d). Analysis of current activation kinetics did not indicate differences between the two conditions (Figure 7c). Interestingly, *I*_k_ current density, activation kinetics, and peak currents were largely overlapping with those found in Non-Tg cultures (Figure 7a and e–g). To further dissect the ROS role in the modulation of 3xTg-AD spontaneous synaptic activity, we investigated changes in [Ca^2+^]_i_ transients in neurons preincubated with NAC (1 mM) for 48 h. NAC-treated 3xTg-AD neurons displayed significant decreases in [Ca^2+^]_i_ oscillations [average spike frequency: 4.682±0.123 (S.E.M.) obtained in 143 neurons from 7 cultures; Figure 8a] when compared with untreated 3xTg-AD cultures (average spike frequency values: 6.312±0.1965 (S.E.M.) obtained in 322 neurons from 14 cultures; *P*<0.001; Figure 8a). NAC treatment did not reduce mean spike frequencies in 3xTg-AD to values observed in Non-Tg neurons (average spike frequency values were 1.958±0.073 (S.E.M.) in 321 neurons from 15 dishes; Figure 8a). No differences were observed in spike amplitudes between 3xTg-AD (average fluorescence values: 85.71±3.165 (S.E.M.) in 321 neurons from 14 cultures) and 3xTg-AD+NAC (average fluorescence values: 69.04±2.663 (S.E.M.) in 143 neurons from 7 cultures; Figure 8b). Interestingly, NAC pre-treatment had no effects on [Ca^2+^]_i_ oscillations in Non-Tg neurons (average spike frequency values: 2.189±0.033 (S.E.M.) obtained in 159 neurons from 7 cultures; Figure 8a), thereby further substantiating a selective role of oxidative stress in the modulation of 3xTg-AD neuron activity.

As previous studies have suggested that NAC may also act independently of its antioxidant properties,^28^ we tested our experimental paradigm with an additional antioxidant molecule. 3xTg-AD and Non-Tg neuronal cultures were therefore incubated for 24 h with the ROS scavenger, Trolox (6-hydroxy-2,5,7,8-tetramethylchroman-2-carboxylic acid), and spontaneous synaptic activity evaluated. Similarly to NAC, Trolox significantly decreased 3xTg-AD neuronal hyperexcitability, thereby supporting the role of oxidative stress as critical mediator of the phenomenon. Compared with untreated 3xTg-AD neurons, Trolox-treated cultures showed a significant decrease in spiking activity (average spike frequency: 1.304± 0.8711 (S.E.M.) obtained in 74 neurons from three cultures; *P*<0.001; Figure 8a). Trolox treatment did not modify spike frequency in Non-Tg neurons (average spike frequency in Trolox-treated Non-Tg cultures: 1.620±0.0871 (S.E.M.) obtained in 127 neurons from four cultures). When considering Trolox effects in Ca^2+^ spike amplitudes we found that, analogously to what observed in NAC-treated cultures, the molecule did not modify spike amplitudes in 3xTg-AD neurons (Figure 8b). However, compared with untreated Non-Tg cultures, NAC- and Trolox-treated Non-Tg neurons showed decreased spike amplitudes (average fluorescence values in NAC-treated neurons: 105± 3.284 (S.E.M.) in 159 cells from seven cultures; and in Trolox-treated neurons: 66.32±2.781 (S.E.M.) in 127 neurons from four cultures; *P*<0.001, Figure 8b).

## Discussion

Our study supports the idea of a causative link between the development of hyperexcitability, increased spontaneous synaptic activity, and the ROS-dependent appearance of conglomerates of dysfunctional Kv2.1 channels. These results are in line with accumulating evidence indicating that AD is characterized by the development of aberrant network activity,^2, 29^ hypersynchrony, epileptiform spiking and compensatory circuit remodeling.^4, 30, 31^

Analysis of spontaneous synaptic activity in our model confirms that 3xTg-AD neurons show a pattern of high [Ca^2+^]_i_ spike frequency that is associated with reduced amplitudes, two phenomena indicative of hyperexcitability.

### KV2.1 conglomeration and activation: the oxidative link

Suggesting a causative role for Kv2.1 channels in the hyperexcitability that we have observed in 3xTg-AD neurons, we found significant changes in channel activity of our AD model. The result can be explained by the high levels of oxidative stress that these neurons are known to face throughout development.^13, 14^ In AD, oxidative stress is a detrimental feature that has been suggested to occur even before the appearance of significant levels of plaque formation, neuropathological alterations or cognitive decline.^29, 32^ Oxidative stress is also strongly enhanced in the brain of preclinical AD models including the 3xTg-AD mouse.^33, 34^ In our experiments, compared with Non-Tg cultures, 3xTg-AD hippocampal neurons were found to be more prone to ROS overproduction in conditions of NMDA receptor activation (Figure 3a and b). ROS are instrumental in promoting Kv2.1 channels oligomerization, a process that favors the formation of conglomerates of dysfunctional channels that, through this step, become resistant to proteolytic cleavage, internalization or endocytosis.^12^ These mechanisms lead to increased Kv2.1 surface expression without a matching increase in channel-mediated whole-cell K^+^ currents. Recent data indicate that when channel density increases on the plasma membrane, non-clustered channels cease to conduct.^35^ In agreement with this model, we found that 3xTg-AD neurons showed increased Kv2.1 oligomerization that pairs with decreased channel conductances (Figures 2 and 4).

These findings are confirmed by a set of complementary results in which we found that, in Non-Tg cultures, GxTx treatment produces an enhanced spontaneous [Ca^2+^]_i_ spike frequency that overlaps with the one observed in 3xTg-AD neurons (Figure 5c and e). In Non-Tg neurons, the same treatment induced a decrease in *I*_k_ currents that is similar to the one observed in 3xTg-AD neurons (Figure 5b). Moreover, GxTx treatment in 3xTg-AD neurons failed to promote any significant modification in *I*_k_ current densities as well as [Ca^2+^]_i_ spike frequency, thereby suggesting that the Kv2.1 channel population present in 3xTg-AD neurons is largely compromised and not susceptible to further pharmacological modulation (Figure 6a and c). The slight increase in [Ca^2+^]_i_ spike amplitudes observed in GxTx-treated 3xTg-AD neurons (Figure 6d) may be the result of compensatory activity of other [Ca^2+^]_i_ regulating systems triggered by the sudden drug-mediated inhibition of residual populations of Kv2.1 channels.

The relationship between high levels of oxidative stress and the appearance of Kv2.1 loss of function is further indicated by the NAC experiments. NAC pre-treatment rescued neuronal activity, decreased [Ca^2+^]_i_ oscillations, and increased normalized *I*_k_ currents, (Figures 7 and 8). Trolox treatment was also able to decrease spontaneous [Ca^2+^]_i_ oscillations (Figure 8). All these findings support a causative role for oxidative stress in setting the stage for Kv2.1-dependent neuronal hyperactivity. We, therefore, propose a sequence of pathogenic events that is centered on ROS-dependent modulation of the plasma membrane distribution of Kv2.1 channels. Lending support to this hypothesis, Kv2.1 immunostaining showed higher levels of plasma membrane appearance and clusterization of the channels (Figure 3). Our immunostaining data show that Kv2.1 are highly restricted on 3xTg-AD somatic and proximal dendritic membranes ^36, 37^ where appear in large clusters (Figure 4), results that are in line with previous studies.^11, 12^

Astrocyte–neuron interactions are central in the modulation of neuronal and synaptic physiology. Kv2.1 channels are located on the soma and principal dendrites of both pyramidal and inhibitory neurons. Furthermore, the channels are strategically present at sites in the close proximity of astrocytic processes. This intercellular distribution promotes a rapid removal of the neuronal K^+^ that is released into the extracellular space upon intense channel activation.^38^ The possibility that such sophisticated regulatory mechanism may be altered in 3xTg-AD neurons represents an intriguing hypothesis that warrants future investigation.

Another important issue concerns the possibility that ROS may affect Kv2.1 activity by employing mechanisms that go beyond channel oxidation. In that respect, previous studies have shown that activity- and calcineurin-dependent Kv2.1 dephosphorylation induces hyperpolarizing shifts. Furthermore, experimental evidence have indicated 16 different Kv2.1 phosphorylation sites, only seven of which are dephosphorylated by calcineurin, thereby indicating multiple mechanisms regulating the channel biophysical properties.^39^

## Conclusions

Our study indicates a scenario where AD-related mutations may promote enhanced ROS generation thereby leading to oxidative-dependent oligomerization and loss of function of Kv2.1 channels, and, ultimately, hyperexcitability. Our model also suggests that interruption of this cycle may offer promising novel therapeutic approaches for AD.

## Materials and methods

### Chemicals and reagents

Fluo-4 AM, HEt and pluronic acid were purchased from Molecular Probe (Invitrogen, Milan, Italy). TTX, NMDA, Glycine, MK-801, CNQX, NAC and Trolox were obtained from Sigma-Aldrich (Milan, Italy). GxTx was purchased from Alomone Lab (Jerusalem, Israel). Tissue culture media and sera were from Gibco (Invitrogen, Milan, Italy). Alexa 488 conjugated anti mouse secondary antibodies and Alexa 546 conjugated anti-rabbit secondary antibodies were from Life Technologies. All other chemicals and reagents were obtained from common commercial sources.

### Animal handling and tissue preparation

All the procedures involving animals were approved by the institutional Ethics Committee (47/2011/CEISA/COM) and performed in accordance with institutional guidelines and national (D.L. n. 116, G.U., suppl. 40, 18 February 1992) and international laws and policies. Groups of 3–4 female mice were housed in colorless cages while male mice were single-housed. Mice were kept on a 12/12 light/dark cycle and had ad libitum access to food and water. All efforts to minimize mice suffering were adopted. Murine hippocampal cultures were prepared from embryonic mice (E16–18). Briefly, hippocampal tissues were dissected in ice-cold, Ca^2+^ free dissecting medium and subsequently minced with forceps. Hippocampal tissue containing medium was then transferred in a 0.25% trypsin solution for 10 min at 37 °C. Hippocampal tissue was then centrifuged at 1300 r.p.m., 4 °C for 5 min. After supernatant removal, pellet was dissociated with a fire-polished glass pipette. Dissociated hippocampal neurons were then re-suspended in Neurobasal medium supplemented with 1 × B27, 5% horse serum, 5% fetal bovine serum, 0.5 mM l-glutamine and 0.2% penicillin/streptomycin. Neurons were then plated on culture plates or dishes previously treated laminin/poly-dl-lysine. A total of 5 *μ*M of cytosine arabinofuranoside was added to the growth medium at 3 to 5 days *in vitro* (DIV) to arrest and inhibit excessive glial proliferation. Media changes were performed by replacing, every three days, 25% of the medium with fresh Neurobasal (a medium that does not contain FBS, HS or B27). Experiments were performed on cultures between 14 and 19 DIV. As far as the cellular composition of our cultures, it should be underlined that the cytostatic treatment was able to halt most of the glial cell replication. However, when we assayed, with anti-GFAP (to detect glial cells) and anti-MAP-2 (microtubule-associated protein 2; to detect neuronal structures) antibodies, the presence of astrocytes in our cultures, a significant amount (around 30%) of these cells was found. Thus, our cultures represent a viable mixture of neurons and supporting glial cells, a physiological setting that allows full interaction between neurons and astrocytes.

### Neuronal culture immunofluorescence

Hippocampal neuronal cultures were fixed for 20 min with ice-cold 4% paraformaldehyde, permeabilized in 0.1% Triton X-100, blocked in 10% of goat serum in phosphate-buffered saline (PBS) and incubated overnight at +4° with anti-Kv2.1 antibody (clone K89/34, dil: 1:200, Neuromab, UC Davis, Davis, CA, USA) together with anti MAP-2 antibody (1:100) in the same blocking solution. Cultures were then stained with species-specific Alexa-conjugated secondary antibodies for 1 h at room temperature in the dark and then mounted on Zeiss Meta confocal microscope. For Kv2.1 clusterization analysis, each neuron was analyzed by evaluating the surface areas occupied by the soma and by Kv2.1 clusters. The two values were then used to obtain a parametric ‘clusterization' index expressed as % value.

### Ca^2+^ imaging and spontaneous [Ca^2+^]_i_ spikes analysis

Spontaneous [Ca^2+^]_i_ spikes were recorded from homogenous neuronal clusters composed of at least 15–25 cells. Neurons were loaded at least 30 min in the dark with the high affinity Ca^2+^ sensitive probe Fluo-4 AM at final concentration of 5 *μ*M plus 0.1% Pluronic F-127 in a HEPES-buffered saline solution (HBSS) composed of (in mM): 20 HEPES, 15 glucose, 120 NaCl, 5.4 KCl, 1.8 CaCl_2_, 0.8 MgCl_2_, 10 NaOH and pH7.4. After loading cells were washed, and incubated in the dark for further 30 min in HCSS. All loading procedures and recordings were carried out at room temperature. No detectable differences in intracellular distribution of dye loading were observed between 3xTg-AD and Non-Tg neurons. Pharmacological stimulations were applied at the time points indicated in text or figures. Drugs were washed out employing a perfusion system synchronized to image acquisition (RSC-200, Bio-logic Science Instrument, Claix, France). Analysis was performed only on neuronal clusters displaying spontaneous synaptic activity within the first minute of the experimental session. Region of interests were manually selected and placed on neuronal somata. Images were obtained using a 16 bit digital EMCCD camera (PhotoEvolve 512; Photometrics, Tucson, AZ, USA) attached to an AxioExaminer upright microscope (Zeiss, Oberkochen, Germany) equipped with a 40 × (NA:1.0) epifluorescence water immersion objective. Light source was provided by a monochromator equipped with a 75 W Xenon lamp (Cairn Instruments, Faversham, UK; excitation, 490 nm; emission, 530 nm). Images acquisition and off-line analysis were performed with Metafluor 7.7 (Molecular Device, Sunnyvale, CA, USA). 16 bit, 515x512 fluorescence images were acquired every 1 s throughout experimental sessions, with the exception of GxTx treatments where images were acquired every 500 ms in order to identify also subtle changes in [Ca^2+^]_i_ spikes frequency after pharmacological challenge. Raw fluorescence values of spontaneous [Ca^2+^]_i_ spikes were normalized and expressed as percentage of baseline fluorescence (% of basal fluorescence). Using a custom made MATLAB code, we calculated the number of peaks (*P*) and mean amplitudes of [Ca^2+^]_i_ spikes. In the final analysis, we took in account only fluorescence values that were 25% larger than baseline.

### ROS measurement

Oxygen radical production was monitored using the oxidation sensitive dye HEt (Ex *λ*: 530±15 nm, Em *λ*: 575–610 nm). Stock HEt (1 mg/ml) was prepared as previously described^40^ in dry DMSO and stored in frozen aliquots for use within eight weeks. Cultures were loaded in the dark with 5 *μ*M HEt in HCSS (45 min, 25 °C). After loading, cultures were washed three times in HCSS and mounted on microscope stage in a static bath of HCSS containing 5 *μ*M HEt. HEt was dissolved in all the solution throughout all the experimental session in order to maintain dye equilibration. Images were acquired by the same imaging setup previously described (see Ca^2+^ Imaging and spontaneous [Ca^2+^]_i_ spikes analysis section). Cells were excited at 530 nm and emission was monitored at >590 nm. To prevent the antioxidant activity of B27, at DIV 5 neuronal cultures were switched to a B27-free Neurobasal medium.

### Electrophysiology

*I*_k_ current recordings were obtained with the whole-cell voltage-clamp configuration.^41^ Cells were mounted on an AxioExaminer microscope, patch pipettes pulled from borosilicate glass tubing (Science Product GmBh, Hofheim, Germany) and heat-polished at the tip to give a resistance of 3–6 MΩ. Electrodes were filled with a intracellular solution composed by (in mM): 10 NaCl, 117 KCl, 2 MgCl_2_, 11 HEPES, 11 ethylene glycol-bis-(*β*-aminoethyl ether)-*N*,*N*,*N*′,*N*′-tetraacetic acid (EGTA), and 1 CaCl_2_, at pH 7.2. Bath solution contained (in mM): 135 NaCl, 5 KCl, 1.2 MgCl_2_, 5 HEPES, 2.5 CaCl_2_, and 10 d-glucose, at pH 7.4. 0.3 *μ*M TTX was continuously applied. All recordings were performed at room temperature (23–25 °C). Holding potential was clamped at −60 mV to reduce the contribution of A-type currents, from this holding value the following voltage protocol was used: 1 s test pulses from −100 to +60 mV in 10 mV steps, followed by a 500 ms tail pulse to −30 mV. Resting membrane potential was recorded using the above mentioned solutions in absence of TTX. Immediately after whole-cell access amplifier was switch from Voltage Clamp to *I*=0 and traces acquired for 10 s in gap-free mode.

A sampling interval of 25 *μ*s/point was used and currents filtered at 5 kHz. Linear components of leak and capacitive currents were canceled using the P/N method. The Nernst K equilibrium potential *E*_K_ was calculated as—79.4 mV. The normalized conductance was plotted against the test potential (*V*) and fitted to a single Boltzmann equation *G*=*G*_max_/(1exp[–(*V*–*G*_1/2_)/*k* ]). Here, *G*_max_ is the maximum conductance, G1/2 is the test potential at which the *I*_K_ channels have a half-maximal conductance, and *k* represents the activation curve slope. In the off-line data analysis, *I*_k_ currents were evaluated at the steady-state amplitude selecting the last 500 ms of the 1 s test pulse. Stimulation, acquisition, and data analysis were performed with pCLAMP 10.0 and Clampfit software and Axopatch 200B amplifier (Molecular Device).

### Western blot analysis

Proteins were extracted from 18 DIV hippocampal cultures. Cultures were washed three times in PBS, scraped and then homogenized in ice-cold lysis buffer (made of 100 mM NaCl, 50 mM Tris–HCl, 40 mM *β*-glycerophosphate, 200 *μ*M sodium orthovanadate, 50 mM NaF, 100 *μ*g/ml phenylmethylsulfonyl fluoride, 10 *μ*g/ml leupeptin, 5 *μ*g/ml pepstatin A, 10 *μ*g/ml benzamidine, 5 mM EDTA, 1% Triton X-100; at pH 7.4). To remove cellular debris, homogenates were centrifuged at 1000 × *g* at 4 °C. Supernatants were then collected and protein concentrations determined by Bio-Rad protein assay (Bio-Rad Laboratories Srl, Segrate, Milan, Italy). Protein containing samples were suspended in Laemmli buffer (containing 8% (w/v) SDS, 10% (v/v) glycerol, 5% (v/v) *β*-mercaptoethanol, 25 mM Tris–HCl, pH 6.5, and 0.003% (w/v) bromophenol blue), boiled for 5 min, and separated by SDS-PAGE on a 10% (w/v) homogeneous slab gel. A total amount of 40 *μ*g of protein, for each sample, were electroblotted onto a nitrocellulose membrane (Amersham Hybond-ECL; GE Healthcare, Milan, Italy). Membranes were then blocked in TBS-T (Tris-buffered saline with 0.1% (v/v) Tween 20) containing 5% (w/v) fat-free milk and incubated overnight with the primary anti-Kv2.1 antibody (clone K39/25 dilution 1:500, NeuroMab, UC Davis). Membranes were then incubated with horseradish peroxidase-conjugated anti-IgG. Signal was detected by chemiluminescence (Pierce ECL Plus; Thermo Scientific, Rockford, USA). Blots were then stripped with 2% (w/v) SDS, 1% (v/v) *β*-mercaptoethanol, in 60 mM Tris–HCl (pH 6.8) for 30 min at 50 °C. Stripped blots were then washed and re-probed with an anti-GAPDH antibody (Millipore, Vimodrone (MI), Italy; dilution 1:5000). Signal was revealed by horseradish-peroxidase-conjugated antibody. Chemiluminescence was recorded using an image acquisition system from Uvitec (Cambridge, UK). All of the antibodies were diluted in TBS-T.

### Statistical analysis

Statistical differences were determined with Student's *t*-test for unpaired data. Statistical significance of *I*–*V* traces was calculated using the Friedman test applied to non-linear regressions. Mann–Whitney test was employed for statistical analysis of immunohistochemistry data. All data are expressed as mean±S.E.M. For multiple comparisons, ANOVA and *post hoc* Bonferroni were used to analyse statistical differences as far as spike frequency and signal amplitude.

## Abbreviations

AD: Alzheimer disease
A*β*: *β*-Amyloid
CNQX: 6-cyano-7-nitroquinoxaline-2,3-dione
DIV: days *in vitro*
GxTx: Guangxitotoxin
HEt: Hydroethidine
*I*_K_: Delayed Rectifier K^+^ Currents
MAP-2: microtubule-associated protein 2
NAC: *N*-acetyl-cysteine
NMDA: *N*-methyl-d-aspartate
PBS: phosphate-buffered saline
ROS: reactive oxygen species
Trolox: 6-hydroxy-2,5,7,8-tetramethylchroman-2-carboxylic acid
TTX: tetrodotoxin
3xTg-AD: triple transgenic mouse model of Alzheimer's disease
Non-Tg: non-transgenic mouse model
