# Supplementary material for: Altered Kv2.1 functioning promotes increased excitability in hippocampal neurons of an Alzheimer's disease mouse model
Source: Cell Death Dis. 2016 Feb 18;7(2):e2100–. doi: 10.1038/cddis.2016.18 (PMC5399189; doi:10.1038/cddis.2016.18)
Supplement: Supplementary Information [file cddis201618x1.docx]

**Supplementary Materials**

**Supplementary Figure 1.**

(**A**) Comparison of resting membrane potentials in 3xTg-AD and Non-Tg neurons. Bar graph shows resting membrane potentials in 3xTg-AD and Non-Tg neurons. No significant statistical differences were found in resting membrane potential values between 3xTg-AD [13 neurons from 3different cultures] and Non-Tg neurons [9 neurons from 3 different cultures (p>0.05) ]. (**B and C**) Representative confocal images of hippocampal neurons from 3xTg-AD cultures before (**B**) and after (**C**) 5 min exposure to 50 μM NMDA + 10 μMGly. Cells were fixed in ice-cold 4% paraformaldehyde 20 minutes after the treatment and stained for MAP2 (blue) and Kv2.1 (Red). Note the de-clusterization of the Kv.2.1 channels after the excitotoxic challenge (inserts in panel **B** and **C**).
