# Supplementary figures and images for: Altered Kv2.1 functioning promotes increased excitability in hippocampal neurons of an Alzheimer's disease mouse model
Source: Cell Death Dis. 2016 Feb 18;7(2):e2100–. doi: 10.1038/cddis.2016.18 (PMC5399189; doi:10.1038/cddis.2016.18)

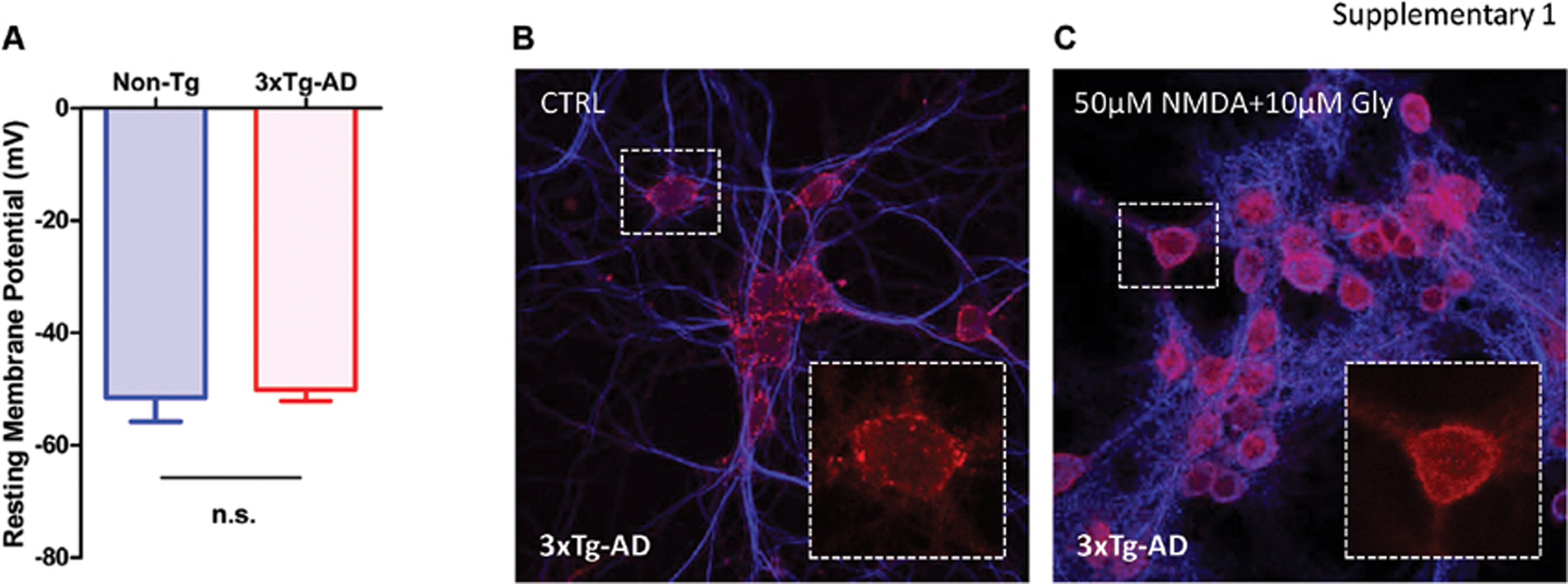

Supplement: Supplementary Figure 1 [file cddis201618x2.tif]
